# Comparative study on the impact of remimazolam and sevoflurane on quality of recovery after transurethral resection of bladder tumor: A randomized controlled noninferiority study

**DOI:** 10.1097/MD.0000000000038962

**Published:** 2024-08-02

**Authors:** Kyoung-Ho Ryu, Sung Hyun Lee, Jae-Geum Shim, Jiyeon Park, Jin Hee Ahn, Suyong Jeon, Eunah Cho

**Affiliations:** a Department of Anesthesiology and Pain Medicine, Kangbuk Samsung Hospital, Sungkyunkwan University School of Medicine, Seoul, Republic of Korea.

**Keywords:** QoR, remifentanil, remimazolam, sevoflurane

## Abstract

**Background:**

Remimazolam is manifested by rapid action, hemodynamic stability, and fast recovery. Our study aimed to investigate whether the quality of recovery (QoR) after remimazolam anesthesia in patients undergoing transurethral resection of bladder tumor, which is predominantly performed in the elderly population, is not inferior to that after conventional anesthesia using sevoflurane.

**Methods:**

Thirty-four patients were randomly allocated into either of group S (n = 17, receiving sevoflurane anesthesia), or group R (n = 17, receiving remimazolam anesthesia). The QoR was assessed by Korean version of QoR-15 questionnaire, on the day before and after the surgery. Scores acquired for each individual item, QoR-15 scores categorized into 5 dimensions (physical comfort, physical independence, psychological support, emotional state, and pain), and overall global score were subjected to comparative analysis. The primary outcome was postoperative global QoR-15, and a noninferiority delta value of 8.0 was employed.

**Results:**

The postoperative global QoR-15 in the group S was 141 (134–146), and in the groups R was 133 (128–142) (*P* = .152). The mean difference of global QoR-15 (group S–group R) was 1.471 (95% confidence interval of −10.204 to 13.146), and the lower 95% confidence interval margin was lower than the noninferiority margin of −8.0. When comparing the QoR-15 sorted by 5 dimensions, pain scored higher in the group S (20 [18–20]) compared to the group R (15 [15–20], *P* = .032).

**Conclusion:**

The postoperative QoR following transurethral resection of bladder tumor was found to be lower in patients anesthetized with remimazolam in comparison to those anesthetized with sevoflurane.

## 1. Introduction

The quality of postoperative recovery holds significant importance in the context of both patient satisfaction and the occurrence of postoperative complications.^[1,2]^ Recently, there has been increasing interest in improving the quality of postoperative recovery, and has led to growing body of research in this area.^[3,4]^ Of particular concern is the recovery process following transurethral resection of bladder tumor (TURBT), a surgical procedure frequently performed on elderly patients.^[5,6]^ Due to their advanced age, these patients are at a higher risk for increased postoperative morbidity, which can be attributed to factors such as polypharmacy, multiple comorbidities, and increased susceptibility to postoperative cognitive dysfunction.^[7,8]^ Furthermore, the placement of a urinary catheter following TURBT can result in catheter-related bladder discomfort (CRBD) which manifests as increased urinary frequency and urgency, thereby potentially exerting negative implications on postoperative recovery.^[7,9,10]^ As such, it is imperative to address these challenges and explore effective strategies to optimize postoperative recovery in TURBT patients.

Remimazolam is a newly introduced ultra-short-acting sedative, which exerts its pharmacological effects by binding to gamma-aminobutyric acid receptors.^[11,12]^ Remimazolam has a rapid onset of action, with a distribution half-life of <1 minute and a metabolic half-life of 10 minutes.^[12]^ It exhibits fast pharmacological action, and is rapidly eliminated through urinary excretion as an inactive metabolite.^[13]^ Additionally, remimazolam is independent of hepatic and renal metabolism and does not accumulate in the tissue, allowing for sustained administration and a quick recovery from anesthesia even with prolonged infusion.^[13]^ Remimazolam demonstrates hemodynamic stability, making it suitable for administration in high-risk populations such as elderly patients and those with cardiovascular or respiratory conditions.^[14–17]^ Furthermore, remimazolam exhibits a fast psychomotor recovery.^[18]^ The rapid pharmacological action, hemodynamic stability, and rapid psychomotor recovery of remimazolam might suggest potential advantages for postoperative recovery following TURBT. However, the overall impact of remimazolam on the quality of recovery (QoR) after TURBT remains unclear.

Therefore, our study aimed to compare the QoR between patients undergoing elective TURBT who received remimazolam anesthesia and those who received sevoflurane anesthesia. Specifically, we designed this study to determine if the QoR following remimazolam anesthesia is noninferior to sevoflurane anesthesia.

## 2. Materials and methods

### 2.1. Ethics

This prospective randomized noninferiority study was performed in a single tertiary hospital from December 2022 to February 2023. Prior to the study, this study was approved by the Institutional Review Board of Kangbuk Samsung Hospital (KBSMC IRB No. 2022-06-043, September 4, 2022), and registered at ClinicalTrials.gov (NCT05356091, first registration date May 2, 2022). Before their study participation, written informed consent was obtained from the study participants. All methods were performed in accordance with the relevant guidelines and regulations as approved by the Institutional Review Board of Kangbuk Samsung Hospital. All study procedures were performed in accordance with the relevant guidelines and regulations.

### 2.2. Study participants

This study included adult patients (above 18 years old) with American Society of Anesthesiologists physical class I–III, who are scheduled for elective TURBT. Exclusion criteria are as follows: patient refusal to participate in the study; unable to understand the consent form (e.g., illiteracy, visual impairment); history of allergy to benzodiazepines; impaired liver, kidney, or cardiac function; pregnancy or breastfeeding patient; drug or alcohol addiction; obesity (body mass index >30 kg/m^2^).

### 2.3. Randomization and blinding

Patients were randomly allocated to group S (sevoflurane) or group R (remimazolam), in a 1:1 ratio. The randomization list was generated using an internet-based randomization algorithm (http://www.randomization.com), in advance. The allocation to each group was determined on the preoperative day 1 in sequential order. The anesthesiologist performed anesthesia according to the allocated group. Throughout the entire study period, the study participants and the researcher collecting data remained blinded to the group assignments.

### 2.4. Anesthetic and study procedure

Premedication was not used in all patients. After entering the operation room, each patient was monitored with noninvasive blood pressure, electrocardiogram, pulse oximetry, surgical pleth index (GE Healthcare, Helsinki, Finland), and electroencephalography (SedLine®; Masimo Corp., Irvine, CA). The intravenous (IV) line was assessed to ensure that the IV catheter was correctly placed without any kinking or obstruction. After checking the patient’s airway, preoxygenation was performed with 100% oxygen via facial mask for 3 minutes.

Anesthetic induction and maintenance were conducted according to the allocated group. For the group S, propofol 1.5 to 2 mg/kg IV was administered. After confirming the loss of consciousness, rocuronium 0.4 mg/kg IVs were given, and ventilated via facial mask with sevoflurane 5 vol% and 100% oxygen. After 90 seconds, i-gel (Intersurgical Ltd, Wokingham, Berkshire, UK) was inserted for airway securement. General anesthesia was maintained with sevoflurane 1.5 to 2.5 vol% to target patient state index (PSI) 30 to 50. For the group R, patients were induced with remimazolam 12 mg/kg/h until achieving loss of consciousness and bolus dose of remifentanil 1 mcg/kg. After loss of consciousness, rocuronium 0.4 mg/kg IV was administered, and the patient was ventilated with 100% oxygen via facial mask. After 90 seconds, i-gel was inserted, and the lungs were mechanically ventilated after confirming proper i-gel insertion. Maintenance of general anesthesia for the group R was performed with continuous remimazolam targeting PSI of 30 to 50, with remifentanil 0.01 to 0.20 mcg/kg targeting for surgical pleth index below 50.

At the end of surgery, sevoflurane for the group S, and remimazolam and remifentanil for the group R were turned off. Neuromuscular blocking agents were reversed with sugammadex 2 mg/kg IV. For the group R flumazenil 0.3 mg IV was additionally administered. I-gel was removed when the patients open their eyes, and breathe regularly. All patients were sent to the postanesthetic care unit.

### 2.5. Outcome measures

The QoR was assessed using the QoR-15 questionnaire, on the day before surgery, and the day after the surgery.^[1,19]^ The QoR-15 questionnaire consists of 15 items, with each item scores from 0 to 15 points. The total score ranges from 0 to 150 points, where a higher score indicates a higher quality of postoperative recovery.

Postoperative pain, CRBD, use of opioids, presence of delirium, and postoperative nausea and vomiting were collected at 0, 1, 6, and 24 hours after surgery. Postoperative pain was evaluated by verbal numerical rating score (VNRS) reported by the patient where 0 represents for no pain and 10 for worst pain imaginable. For postoperative pain management, ketorolac 30 mg was administered intravenously when the postoperative pain score was between 4 and 6 on the VNRS. If the VNRS score was 6 or higher, 50 mg of tridol was administered intravenously. CRBD was assessed by rating from 1 to 4 as follows: none: does not complain of CRBD on questioning, mild: reports only on questioning, moderate: reports without questioning without behavior responses, severe: reports without questioning accompanied by behavior responses.^[7]^ The incidence of opioid use administered during the postoperative periods of 0–1, 1–6, and 6–24 hours was investigated. The presence of postoperative delirium was assessed using the confusion assessment method score as used in the prior study. This score evaluates (1) acute and fluctuating changes in mental status, (2) inattention, (3) disorganized or incoherent thinking, and (4) altered level of consciousness. Postoperative delirium is considered present if there are (1) and (2), along with either (3) or (4), or both.^[7]^

Duration of operation is defined by the time from the cystoscope insertion to foley insertion. Duration of anesthesia was defined by the time from the start of anesthetic induction to discharging the patient to the postanesthetic care unit. Time to remove i-gel was defined by the time from the anesthetic drug discontinuation to i-gel removal.

### 2.6. Sample size calculation

This noninferiority study was aimed to investigate whether QoR after remimazolam anesthesia is noninferior to that of sevoflurane anesthesia, evaluated with QoR-15 questionnaire. We aimed to investigate whether the postoperative global QoR-15 of remimazolam was not inferior to that of sevoflurane. The noninferiority delta was set based on the prior study showing that minimal clinically important difference for the QoR-15 to be 8.0.^[20]^ Considering the noninferiority delta, the sample size was calculated to test the null hypothesis H_0_: −8.0 ≥ μ_0_–μ_1_ versus H_1_: −8.0 < μ_1_–μ_0_, while μ_1_ stands for the mean QoR-15 score of remimazolam, and μ_0_ for that of sevoflurane. With a power (1 − β) of 0.80 and a significance level of 0.05, and a dropout rate of 20%, a total of 36 participants, with 18 in each group, were required for the study using the authors’ preliminary data (unpublished) collected from consecutive 10 patients, the global QoR-15 score after sevoflurane anesthesia was 104.4 ± 12.1.

### 2.7. Statistical analysis

Data are expressed as mean ± standard deviation, median (interquartile range), numbers (%), or 95% confidence interval (CI). Continuous variables were compared using the Student *t* test for the normally distributed data, or the Mann–Whitney *U* test for the nonnormally distributed data. To compare preoperative and postoperative QoR scores, the Wilcoxon signed rank test was conducted. Categorial variables were compared by using the Chi-square test or Fisher exact test, as appropriate. It is considered noninferior when the lower margin of 2-sided 95% CI was above the noninferiority margin of −8.0. *P*-value < .05 was considered to be statistically significant. R [version 4.2.2 (http://www.R-project.org); R Foundation for Statistical Computing, Vienna, Austria] was used for the statistical analyses.

## 3. Results

**Figure 1.**
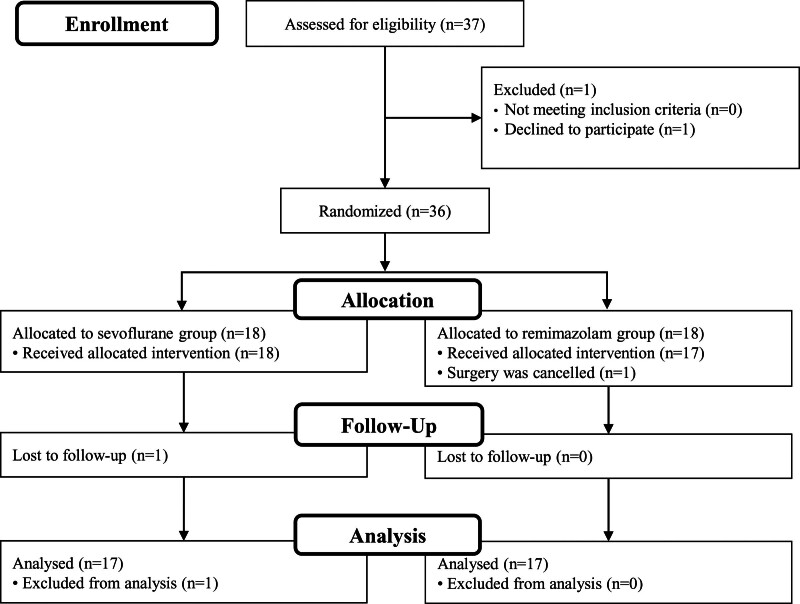
CONSORT diagram of the study.

This study was performed from December 2021 to September 2022. Thirty-seven patients were assessed for eligibility for the study participation, and 1 patient declined to participate. Thirty-six patients were randomly allocated to group S (n = 18), and group R (n = 18). Among the 18 patients in the group R, 1 patient was dropped off because the surgery was canceled. One patient in the group S was lost to follow-up. Therefore, 17 patients in each group were included in the final analysis (Fig. 1).

The characteristics of the study population and surgical data are described in Table 1. There was no difference between the groups as to the ate, sex, height, weight, body mass index, American Society of Anesthesiologists PS, urethral catheter size, tumor sate, tumor size, and multiplicity.

**Table 1:** Demographic characteristics and surgical data of the study population.

Variables	Overall (N = 34)	Group S (N = 17)	Group R (N = 17)	*P*-value
Age, y	68.97 ± 10.97	66.18 ± 11.95	71.76 ± 9.42	.140
Sex				.396
Male	27 (79.4)	15 (88.2)	12 (70.6)	
Female	7 (20.6)	2 (11.8)	5 (29.4)	
Height, cm	164.3 (159.9–170.4)	162.7 (160.0–168.5)	166.6 (158.3–171.3)	.986
Weight, kg	66.5 ± 7.9	66.5 ± 8.8	66.5 ± 7.2	>.999
BMI, kg/m^2^	25.3 (23.5–26.5)	25.1 (23.4–26.4)	25.4 (23.8–26.5)	.836
ASA PS				.620
1	8 (23.5)	5 (29.4)	3 (17.6)	
2	16 (47.1)	8 (47.1)	8 (47.1)	
3	10 (29.4)	4 (23.5)	6 (35.3)	
Foley catheter size, Fr	18 (18–22)	18 (18–22)	18 (18–22)	.728
Tumor stage				.475
No evidence of cancer	8 (23.5)	4 (23.5)	4 (23.5)	
Noninvasive papillary carcinoma	15 (44.1)	9 (52.9)	6 (35.3)	
Carcinoma in situ	1 (2.9)	1 (5.9)	0 (0)	
Invading lamina propria	8 (23.5)	3 (17.6)	5 (29.4)	
Invading muscularis propria	2 (5.9)	0 (0)	2 (11.8)	
Tumor size				.174
<1 cm	1 (3.0)	1 (5.9)	0 (0)	
1–3 cm	26 (78.8)	15 (88.2)	11 (68.8)	
3–5 cm	4 (12.1)	1 (5.9)	3 (18.8)	
>5 cm	2 (6.1)	0 (0)	2 (12.5)	
Multiplicity				>.999
Single	16 (48.5)	8 (47.1)	8 (50.0)	
Multiple	17 (51.5)	9 (52.9)	8 (50.0)	

Data are presented by mean ± SD, median (interquartile range), and numbers (%).

ASA PS = American Society of Anesthesiology Physical Status, BMI = body mass index, SD = standard deviation.

Intraoperative and postoperative data are presented in the Table 2. Postoperative pain was not different between the 2 groups at 0 (*P* = .529), 1 (*P* = .723), 6 (*P* = .653), and 24 hours (*P* = .281). The intensity of CRBD showed no difference throughout the study period. The incidence of postoperative opioid use was 0%, 0%, 23.5%, and 0% at 0, 1, 6, 24 hours in the group S, and 0%, 5.9%, 35.3%, and 5.9% in the group R. The incidence of postoperative delirium and postoperative nausea and vomiting was not different between the groups. Although operation duration was not different (15 [10–25] minutes in the group S, vs 15 [10–15] minutes in the group R, *P* = .056), anesthesia duration was longer in group R (39 [32–53] minutes) compared to that in group S (29 [23–37] minutes, median difference: 10 [95% CI: 2–21], *P* = .011). Time to i-gel removal was not different between the 2 groups.

**Table 2:** Intraoperative and postoperative data.

Variables	Group S (N = 17)	Group R (N = 17)	Difference (95% CI)	*P*-value
Postoperative pain, VNRS				
0 h	0 (0–0)	0 (0–0)	0 (0–0)	.529
1 h	0 (0–3)	0 (0–2)	0 (−2 to 0)	.723
6 h	0 (0–2.3)	0 (0–1)	0 (−2 to 1)	.653
24 h	0 (0–0.3)	0 (0–2.3)	0 (0–1)	.281
CRBD				
0 h	2 (1.8–3)	2 (1–2.3)	0 (−1 to 0)	.216
1 h	2 (2–2)	2 (2–3)	0 (0–1)	.131
6 h	2 (2–2)	2 (2–2)	0 (0–0)	.685
24 h	2 (1–2)	1 (1–2)	0 (−1 to 0)	.176
Opioid use				
0–1 h	0 (0.0)	1 (5.9)	−0.059 (−0.059 to 0.053)	>.999
1–6 h	4 (23.5)	6 (35.3)	−0.118 (−0.3795 to 0.1857)	.708
6–24 h	0 (0)	1 (5.9)	−0.059 (−0.059 to 0.053)	>.999
Delirium				
0 h	1 (5.9)	1 (5.9)	−0.059 (−0.059 to 0.053)	>.999
1 h	0 (0)	1 (5.9)	−0.059 (−0.059 to 0.053)	>.999
6 h	0 (0)	0 (0)	0 (0–0)	N/A
24 h	0 (0)	0 (0)	0 (0–0)	N/A
PONV				
0 h	0 (0)	0 (0)	0 (0–0)	N/A
1 h	0 (0)	0 (0)	0 (0–0)	N/A
6 h	0 (0)	2 (11.8)	−0.118 (−0.118 to 0.035)	.485
24 h	0 (0)	0 (0)	0 (0–0)	N/A
Operation duration, min	15 (10–15)	15 (10–30)	5 (0–15)	.056
Anesthesia duration, min	29 (23–37)	39 (32–53)	10 (2–21)	.011*
Time to remove i-gel, min	4 (2–5)	3 (2–4)	−1 (−2 to 1)	.340

Data are presented by median (interquartile range) and numbers (%).

CRBD = catheter-related bladder discomfort, N/A = not available, PONV = postoperative nausea and vomiting, VNRS = verbal numerical rating score.

* *P*-value < .05.

There was no significant difference in QoR-15 scores between the 2 groups, whether observed for individual items or global scores. Baseline preoperative global QoR-15 score was not different between the group S [144 (122–150)] and group R [139 (132–144), *P* = .489]. Postoperative global QoR-15 was 141 (134–146) in the group S, and 133 (128–142) in the group R (*P* = .152). When comparing preoperative and postoperative scores within each group, a decrease in score for 5th item was observed in group S (10 [10–10] preoperative vs 10 [8–10] postoperative, *P* = .017), while an increase in score for 7th item was noted in group R (10 [5–10] preoperative vs 10 [10–10], *P* = .034) (Table 3). When the postoperative QoR-15 scores were divided into 5 dimensions, there was no statistically significant difference between the 2 groups in the physical comfort (*P* = .281), physical independence (*P* = .671), and emotional state (*P* = .759). Among the 5 dimensions, psychological support in group S showed 20 (20–20), while it was 20 (20–20) in group R, with median difference and 95% CI of 0 (0–0), *P* = .036. Scores in the pain dimension were higher in group S (20 [18–20]) compared to that in group R (15 [15–20], median difference −2, 95% CI: −5 to 0, *P* = .032) (Table 4).

**Table 3:** Preoperative and postoperative QoR-15 score.

Variables	Group S (N = 17)			Group R (N = 17)				Median difference (95% CI)†	SMD†	*P*-value†	*P*-value‡
	Pre	Post	*P*-value§		Pre	Post	*P*-value§				
1. Able to breathe easily	10 (10–10)	10 (10–10)	.684		10 (10–10)	10 (10–10)	.317	0 (0–0)	0.457	.268	.074
2. Been able to enjoy food	10 (10–10)	10 (8–10)	.609		10 (10–10)	10 (10–10)	.715	0 (0–0)	<0.001	.374	.378
3. Feeling rested	10 (8–10)	10 (9–10)	.672		10 (10–10)	10 (9–10)	.116	0 (0–0)	0.193	.917	.074
4. Have had a good sleep	10 (9–10)	8 (4–10)	.345		10 (7–10)	10 (7–10)	.905	1 (0–4)	0.455	.150	.973
5. Able to look after personal toilet and hygiene unaided	10 (10–10)	10 (8–10)	.017*		10 (10–10)	10 (10–10)	.063	0 (0–1)	0.190	.372	>.999
6. Able to communicate with family or friends	10 (9.75–10)	10 (10–10)	.357		10 (10–10)	10 (10–10)	>.999	0 (0–0)	0.594	.036*	.786
7. Getting support from hospital doctors and nurses	10 (10–10)	10 (10–10)	.273		10 (5–10)	10 (10–10)	.034*	0 (0–0)	0.456	.151	.540
8. Able to return to work or usual home activities	10 (10–10)	10 (8–10)	.066		10 (10–10)	10 (8–10)	.244	0 (0–0)	0.135	.915	>.999
9. Feeling comfortable and in control	10 (10–10)	10 (10–10)	.414		10 (10–10)	10 (10–10)	.109	0 (0–0)	0.628	.074	.634
10. Having a feeling of general well-being	8 (5–10)	10 (8–10)	.046		9 (7–10)	10 (8–10)	.372	0 (0–0)	0.072	.915	.496
11. Moderate pain	10 (10–10)	10 (8–10)	.127		10 (9–10)	7 (5–10)	.164	−2 (−5 to 0)	0.604	.089	.496
12. Severe pain	10 (10–10)	10 (10–10)	.180		10 (10–10)	10 (10–10)	.068	0 (0–0)	0.356	.351	>.999
13. Nausea or vomiting	10 (10–10)	10 (10–10)	.180		10 (10–10)	10 (10–10)	.317	0 (0–0)	0.058	.600	>.999
14. feeling worried or anxious	10 (8–10)	9 (6–10)	.351		8 (5–10)	10 (7–10)	.191	0 (0–2)	0.114	.360	.131
15. Feeling sad or depressed	10 (8–10)	10 (9–10)	.750		10 (10–10)	10 (9–10)	.889	0 (0–0)	0.024	.740	.474
Global QoR-15 score	144 (122–150)	141 (134–146)	.712		139 (132–144)	133 (128–142)	.605	−5 (−13–4)	0.090	.152	.489

Data are presented by median (interquartile range).

QoR = quality of recovery, SMD = standard median difference.

† Compared postoperative variables between groups.

‡ Compared preoperative variables between groups.

§ Compared preoperative versus postoperative variables within each group.

* *P*-value < 0.05.

**Table 4:** Postoperative QoR-15 score sorted by each dimension.

	Group S (N = 17)	Group R (N = 17)	Median difference (95% CI)	SMD	*P*-value
Physical comfort	44 (42–50)	46 (44–50)	1 (−1 to 6)	0.236	.281
Physical independence	19 (18–20)	20 (15–20)	0 (−2 to 2)	<0.001	.671
Psychological support	20 (20–20)	20 (20–20)	0 (0–0)	0.645	.036*
Emotional state	38 (35–40)	39 (33–40)	0 (−2 to 3)	0.187	.759
Pain	20 (18–20)	15 (15–20)	−2 (−5 to 0)	0.591	.032*

QoR = quality of recovery, SMD = standard median difference.

Data are presented by median (interquartile range).

* *P*-value < .05.

**Figure 2.**
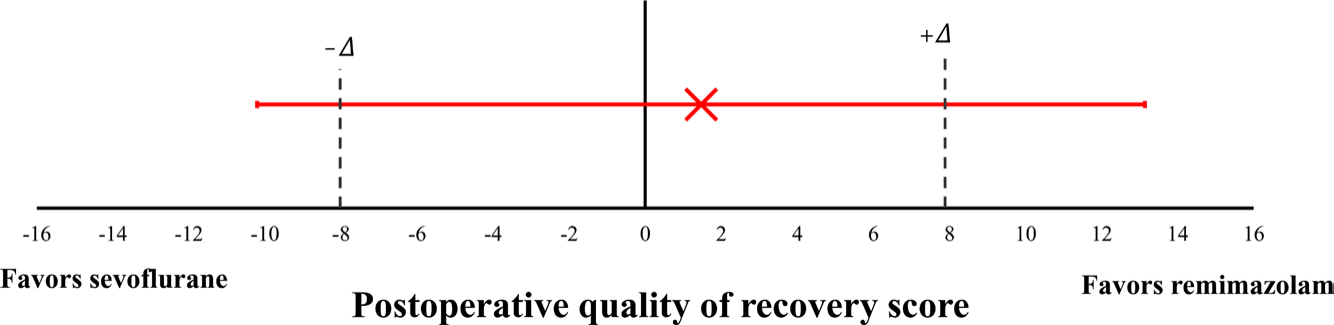
Noninferiority diagram of the mean difference of postoperative global QoR-15 score between the sevoflurane group and the remimazolam group. The dashed line represents the noninferiority margin (𝛥 = −8). The error bars indicate the 95% CI of the postoperative total QoR-15 difference (group R–group S). The diagram describes inferiority of the remimazolam compared to sevoflurane. CI = confidence interval.

The difference in means of postoperative global QoR-15 between group S and group R was 1.47 (95% CI: −13.15 to 10.20), and the lower confidence limit was below the noninferiority margin of −8.0 (Fig. 2).

## 4. Discussion

Our study aimed to investigate the impact of anesthetic agent on postoperative recovery in patients undergoing TURBT, which is characterized by a relatively short operation duration and minimal stimulation. Considering its rapid onset and short duration of action, remimazolam was expected to be a suitable choice for TURBT. Consequently, we hypothesized that the impact of remimazolam on postoperative recovery after TURBT would not be inferior to that of the conventional sevoflurane anesthesia. However, according to our study results, postoperative QoR-15 exhibited a mean difference of 1.471 (95% CI of −10.204 to 13.146), with the lower 95% CI encompassing the noninferiority delta of −8.0. This implies that the QoR following remimazolam anesthesia is inferior to that observed after sevoflurane anesthesia.

In our study, the main anesthetic agents used were remimazolam and sevoflurane. In our hospital, sevoflurane alone is generally used as a standard anesthetic method for TURBT. Sevoflurane being an inhaled anesthetic gas requires IV induction agents, and we commonly use propofol 1 to 1.5 mg/kg for this purpose. Hence, sevoflurane group, as a control, was administered the standard clinical procedure of our hospital, which involved employing sevoflurane as the sole agent following induction with IV propofol. Given that an opioid is necessary to sustain general anesthesia in cases where remimazolam is employed as the primary anesthetic agent, we incorporated remifentanil in conjunction with remimazolam within the experimental group.^[21]^

In recent studies, there has been growing focus on investigating the QoR associated with remimazolam.^[22–24]^ Zhao et al^[25]^ demonstrated that QoR-15 after thoracoscopic laparoscopic radical esophagectomy in elderly patients at postoperative day 1 was higher in the remimazolam group showing 114 compared to 106 in the propofol group. Lee et al^[22]^ showed no difference in the QoR-15 at postoperative day 1 (125 in the remimazolam group vs 129 in the propofol group, *P* = .571) in patients undergoing open thyroidectomy, although remimazolam caused faster eye opening and extubation time. Kim et al reported that QoR-40 score was not different between remimazolam (176.7 ± 12.5) compared to propofol (177.1 7 ± 13.4, *P* = .836). In 1 noninferiority study done by Choi et al^[24]^, QoR-15 after remimazolam anesthesia (111.2 ± 18.8) was noninferior to that after propofol anesthesia (109.1 ± 18.9) in female patients after thyroid surgery. These studies were conducted on a mid-aged population and had longer surgical durations compared to our study. Our study was conducted on patients with bladder cancer undergoing elective TURBT. Bladder cancer is more prevalent with increasing age and is 3 to 4 times more common in males than in females.^[5]^ Thus, the participants in our study also had an average age of 68.97 ± 10.97 years old, and males comprising 79.4%. Therefore, we guess that the differences in study populations compared to other studies might have led to different outcomes.

Our study was designed to administer anesthetic agent (sevoflurane or remimazolam) targeting a PSI range of 30 to 50. Given its familiarity and extensive clinical experience, lowering the sevoflurane to the minimum concentration to achieve a PSI range of 30 to 50 was not challenging. On the other hand, remimazolam was administered at 1 mg/kg/h upon loss of consciousness as recommended by the manufacturer, and the dosage was maintained if the PSI was well maintained. However, due to the lack of sufficient background research on how low remimazolam can be safely maintained, it was difficult to reduce the dosage below the recommended dosage. Given the susceptibility of the elderly to remimazolam, it seems reasonable to consider using lower dosage.^[15,26]^ Moreover, taking into account amnesic effect of remimazolam, and potential synergistic effect with remifentanil, it is anticipated that lower dosage could be administered.^[27]^ However, a comprehensive explanation for the connection between administering excessive remimazolam and the resulting low QoR-15 scores remains uncertain.

Prior studies have predominantly compared QoR between total IV anesthesia using propofol and remimazolam.^[22–24]^ Therefore, these studies have incorporated remifentanil equally in both groups and are designed to specifically compare the influence of the anesthetic agents on the QoR score. In our study, remifentanil was administered only in the remimazolam group. We initially considered not using remifentanil in the remimazolam in order to compare the pure effect of the main anesthetic agents on the QoR. However, remimazolam without remifentanil could not maintain the targeted PSI levels, leading to difficulties in maintaining anesthesia effectively. This is due to the lack of analgesic properties in benzodiazepines, necessitating the use of opioids to block surgical stress.^[28]^ However although, remifentanil was used in our study, the average dosage used in our study was 0.1 mcg/kg/min, which does not exceed the dose level known to increase opioid-induced hyperalgesia (infusion rate above 0.25 mcg/kg/min).^[29]^ Furthermore, TURBT does not manifest severe postoperative pain.^[7]^ Therefore, we believe that using remifentanil would not affect our study outcomes.

In our study, the pain dimension score of the QoR-15 questionnaire was lower for the group R compared to the group S (20 [18–20] in the group S vs 15 [15–20] in the group R, *P* = .032). While there were no significant differences in postoperative pain assessed by VNRS at 0 (*P* = .529), 1 (*P* = .723), 6 (*P* = .653), and 24 hours (*P* = .281). The possible reasons for this discrepancy can be considered as follows. First, the QoR-15 questionnaire assesses the frequency of pain experienced over the past 24 hours (from “none of the time” to “all of the time”), while VNRS measures the severity of pain at specific time points. This methodological difference may account for the discrepancy in pain scores between the 2 groups. Second, the timing of opioid administration relative to pain assessment could have influenced the VNRS pain scores. Since opioids were likely administered before the pain assessments at 1, 6, and 24 hours, the recorded pain severity might have been lower due to the analgesic effect, potentially masking true pain levels.

QoR-15 evaluates mental well-being by dividing it into 2 dimensions: psychological support and emotional state. In our study, the score of the psychological dimension of QoR-15 in group R was significantly higher than in group S. This was consistent with previous studies where remimazolam scored higher in psychological support compared to propofol.^[24,30]^ This is explained by benzodiazepines affecting nonrapid eye movement sleep, thereby improving sleep quality.^[30]^ However, these studies compared remimazolam and propofol, not remimazolam and sevoflurane anesthesia. Moreover, they did not observe direct effects of remimazolam on postoperative sleep. Therefore, further research is needed on the relationship between remimazolam, sleep, and the psychological support dimension score.

There are several limitations in our study. First, we administered remifentanil only in the remimazolam group. Therefore, the outcomes might be differed if remifentanil had been utilized in the sevoflurane group as well. The purpose of our study was to compare the conventional method of anesthesia for TRUBT using sevoflurane with total IV anesthesia using remimazolam. In our clinical experience, opioid is unnecessary for TURBT anesthesia with sevoflurane. Therefore, adding opioid analgesic to the sevoflurane group for the sake of this study was ethically impermissible. Consequently, future follow-up study incorporating remifentanil in both groups will be needed. Second, we assessed the postoperative outcomes only up to 24 hours after surgery. It was because the patients usually recover within a postoperative day 1, and generally discharged on the second day after surgery. Hence, conducting outcome assessments for several days after surgery might have yielded different results.

In conclusion, postoperative QoR-15 was lower after total IV anesthesia using remimazolam compared to volatile anesthesia with sevoflurane in elderly patients undergoing elective TURBT.

## Author contributions

**Conceptualization:** Kyoung-Ho Ryu.

**Data curation:** Kyoung-Ho Ryu, Jae-Geum Shim, Jin Hee Ahn, Eunah Cho.

**Writing—original draft:** Kyoung-Ho Ryu, Jiyeon Park, Jin Hee Ahn, Eunah Cho.

**Formal analysis:** Sung Hyun Lee, Jin Hee Ahn.

**Methodology:** Sung Hyun Lee, Jae-Geum Shim, Eunah Cho.

**Writing—review & editing:** Sung Hyun Lee, Jiyeon Park, Suyong Jeon, Eunah Cho.

**Investigation:** Jae-Geum Shim, Eunah Cho.

**Validation:** Jae-Geum Shim.

**Funding acquisition:** Eunah Cho.
